# The impact of cannabis legalization and decriminalization on acute poisoning: A systematic review

**DOI:** 10.1111/add.16280

**Published:** 2023-07-26

**Authors:** Sara Allaf, Jessy S. Lim, Nicholas A. Buckley, Rose Cairns

**Affiliations:** ^1^ Faculty of Medicine and Health, School of Pharmacy The University of Sydney Sydney NSW Australia; ^2^ New South Wales Poisons Information Centre The Children’s Hospital at Westmead Sydney NSW Australia; ^3^ Faculty of Medicine and Health, School of Medical Sciences, Biomedical Informatics and Digital Health The University of Sydney Sydney NSW Australia

**Keywords:** Cannabidiol, cannabis, decriminalization, legalization, marijuana, poisoning

## Abstract

**Background and Aims:**

Many countries have recently legalized medicinal and recreational cannabis. With increasing use and access come the potential for harms. We aimed to examine the effect of cannabis legalization/decriminalization on acute poisoning.

**Methods:**

A systematic review and meta‐analysis registered with PROSPERO (CRD42022323437). We searched Embase, Medline, Scopus and Cochrane Central Register of Controlled Trials from inception to March 2022. No restrictions on language, age or geography were applied. Abstracts from three main clinical toxicology conferences were hand‐searched. Included studies had to report on poisonings before and after changes in cannabis legislation, including legalization and decriminalization of medicinal and recreational cannabis. Where possible, relative risk (RR) of poisoning after legalization (versus before) was calculated and pooled. Risk of bias was assessed with ROBINS‐I.

**Results:**

Of the 1065 articles retrieved, 30 met inclusion criteria (including 10 conference abstracts). Studies used data from the United States, Canada and Thailand. Studies examined legalization of medicinal cannabis (*n* = 14) and decriminalization or legalization of recreational cannabis (*n* = 21). Common data sources included poisons centre records (*n* = 18) and hospital presentations/admissions (*n* = 15, individual studies could report multiple intervention types and multiple data sources). Most studies (*n* = 19) investigated paediatric poisoning. Most (*n* = 24) reported an increase in poisonings; however, the magnitude varied greatly. Twenty studies were included in quantitative analysis, with RRs ranging from 0.81 to 29.00. Our pooled estimate indicated an increase in poisoning after legalization [RR = 3.56, 95% confidence interval (CI) = 2.43–5.20], which was greater in studies that focused on paediatric patients (RR = 4.31, 95% CI = 2.30–8.07).

**Conclusions:**

Most studies on the effect of medicinal or recreational cannabis legalization/decriminalization on acute poisoning reported a rise in cannabis poisoning after legalization/decriminalization. Most evidence is from US legalization, despite legalization and decriminalization in many countries.

## INTRODUCTION

Many countries have moved to decriminalize or legalize medical and recreational use of cannabis in the past three decades [1]. Decriminalization involves removal of criminal consequences for possession of small quantities of cannabis, while legalization involves removing penalties and allowing for sale and possession of cannabis [2]. In 1996, California became the first state in the United States (US) to legalize cannabis for medical use and possession. This was later implemented in some other states [3]. Currently, medicinal cannabis use is legal in the District of Colombia and 37 states in the US [3]. From these 37 states, 22 have also legalized recreational cannabis [4]. Access to medical marijuana became legal in Canada in 1999 [5]. Recreational use of marijuana has been legal in Canada since 2018 [6]. Luxembourg has recently legalized recreational cannabis, and cannabidiol (CBD) products containing low concentrations of tetrahydrocannabinol (THC) are available in many European countries [7]. In Australia, the Therapeutic Goods Administration legalized the medical use of cannabis and THC in 2016 [8]. Medicinal marijuana was legalized in Thailand in 2019 [9].

Medicinal cannabis legislation expands treatment options for conditions including refractory epilepsy, while recreational cannabis legislation aims to allow safe use while reducing criminal activity and use of other drugs including synthetic cannabinoid receptor agonists (SCRAs). However, these changes can also have negative public health impacts. For example, recreational cannabis legislation in the US has been associated with a rise in motor vehicle accidents, alcohol and opioid abuse [10]. Overall hospitalizations for cannabis and cannabinoid hyperemesis syndrome also increased in Colorado after legalization [11]. The prevalence of cannabis use has increased in US states where medicinal cannabis is legal [12].

With increasing use and availability of cannabis in the community comes the risk of harms, including overdose and poisoning. Cannabis has a wide safety margin in adults; however, overdose can cause central nervous system (CNS) excitation, hallucinations, psychosis, CNS depression, bradycardia and dysrhythmias [13, 14]. Even when used as intended, cannabis use increases the risk of acute coronary syndrome in the hours following consumption [15]. It is more likely to cause severe toxicity in children, manifesting as CNS and respiratory depression, coma, seizures and apnoea [16]. Among the individual cannabinoids, THC is considered more toxic than CBD; however, CBD can cause gastrointestinal, neurological and hepatic symptoms.

Increased prevalence of cannabis poisoning has been observed following legalization [17, 18]. Studies on poisoning typically use data from poisons centres. Poisons centre exposure calls can be triggered for a variety of reasons, including paediatric exposures (e.g. a child accessing edible marijuana products), a person experiencing signs/symptoms of toxicity following therapeutic or recreational use, dosing errors with medicinal marijuana and as an agent in deliberate self‐harm overdoses. Poisons centre coding of substances involved in exposures is typically extremely accurate, as it is coded by the poisons specialist handling the call. However, poisons centres underestimate the rates of exposures, as calls are voluntary. Other studies use hospital presentation or admission data, with reasons for presentation likely to be similar to reasons for calling a poisons centre. There are limitations in how well hospital data collects specific substances involved in poisonings, particularly with recreational drugs [19]. A previous study identified a 34% increase in poison centre calls for cannabis exposure per year following legalization in Colorado [20]. Similarly, emergency department presentations due to cannabis toxicity increased from 1.2 to 2.3 per 100 000 population after legalizing recreational cannabis [20].

We conducted a systematic review to investigate the effect of changing cannabis legalization and decriminalization on poisoning. Specifically, we aimed to evaluate the effect of the legalization of medicinal cannabis, and legalization or decriminalization of recreational cannabis, on acute poisoning with cannabis and cannabinoids.

## METHODS

### Search strategy and data sources

We searched Embase (11 March 2022), Medline, Scopus and Cochrane Central Register of Controlled Trials (CENTRAL) (14 March 2022) without language restrictions from inception until March 2022. Our search strategy focused on three main concepts: cannabinoids, legalization/decriminalization (intervention) and poisonings (outcome). The full search strategy is available in Supporting Information. We performed forwards and backwards citation chaining of included papers to search for additional relevant papers, with the final citation chaining completed in August 2022. We examined grey literature by hand‐searching conference abstracts from the annual conferences of the three main international clinical toxicology societies: Asia Pacific Association of Medical Toxicology (APAMT), the European Association of Poisons Centres and Clinical Toxicologists (EAPCCT) and the American Academy of Clinical Toxicology (AACT) for the past 5 years. This review was registered with PROSPERO (CRD42022323437); a brief protocol is accessible on the PROSPERO website. One amendment has been made to the pre‐registered protocol: we removed SCRAs from the inclusion criteria, as they are not subject to similar legislative changes as cannabis, THC and CBD.

### Inclusion and exclusion criteria

We included studies that investigated the changes in cannabinoid poisoning/exposure frequency or rates following cannabis legalization/decriminalization. Cohort studies, observational studies, repeated cross‐sectional and interrupted time‐series (ITS) studies with pre‐and post‐intervention data sets were included. We focused on acute poisoning, and included studies that reported acute psychosis and acute cardiovascular events. There were no restrictions on age.

We excluded studies that reported on trends in SCRAs only, animal poisoning, chronic psychotic illness, hyperemesis syndrome, chronic cardiovascular disease, cancer and *in‐utero* exposures/developmental toxicity. Studies that discussed the legalization of cannabis but did not report trends in cannabinoid poisoning and those that reported change in cannabinoid poisoning without cannabinoid legalization/decriminalization were excluded.

### Selection of studies

We exported studies into Covidence software (Veritas Health Innovation, Melbourne, Australia) and removed duplicates. Two authors (S.A. and J.L.) independently screened the titles and abstracts for eligibility, and irrelevant articles were removed. Conflicts were discussed with a third author (R.C.). Articles that passed the title and abstract screening were reviewed for inclusion criteria by full‐text screening by two independent authors. A third author (R.C.) resolved conflicts between reviewers.

### Data extraction

Data extraction was performed by two independent authors (S.A. and J.L.) with a structured data collection form. Data extracted included study geographic location, population characteristics, study design, time‐period examined, year and details of the intervention, data sources, type of cannabinoid poisoning (CBD, THC, both), trends in cannabinoid poisoning (poisoning cases/incidence before and after the intervention), median age, clinical effects, clinical outcomes and any other impacts of the legalization or decriminalization.

### Synthesis methods

Studies were stratified according to intervention type (decriminalization versus legalization; medicinal versus recreational use), study population (children/adolescents versus adults), data source (poisons centre versus hospital records) and literature type (grey literature versus peer‐reviewed original research). Studies were eligible for quantitative synthesis if they provided raw counts or population rates. Raw counts were converted to population rates using publicly available population data. Relative risk (RR) was calculated as the rate of cannabis poisoning after the legalization/decriminalization divided by the rate of cannabis poisoning before the legalization/decriminalization. We calculated RR, confidence intervals (CIs) and heterogeneity (*I* ^2^) in Microsoft Excel using the MetaXL tool version 5.3 (www.epigear.com), using the random effects model.

We performed subgroup analysis based on characteristics of studies that we hypothesized may impact on estimates and contribute to heterogeneity. We examined legislation relating to medicinal use versus recreational use, as recreational legislation may result in more widespread use and therefore poisonings. We compared age groups (paediatric versus all ages), as children are at most risk of severe poisoning from cannabinoids. We stratified by data source (poisons centre versus hospital presentations/admissions data), as hospital data are likely to represent a more severe subset of exposures. We also looked for differences by study type comparing the more robust peer‐reviewed literature with grey literature (conference abstracts). Where possible, we also performed stratification analysis, e.g. where one paper included data from poisons centre and hospital events, these ‘substudies’ were stratified accordingly.

GraphPad Prism 9.4 (GraphPad Software, San Diego, CA, USA) was used for data visualization.

### Risk of bias

Two authors (S.A. and J.L.) assessed each study for quality and potential bias using the ROBINS‐I (risk of bias in non‐randomized studies of interventions) tool [21]. For the ‘risk of bias due to confounding’ domain, simple pre–post studies were classified as high risk of bias. Studies that presented simple pre–post data with some quantification of pre‐intervention trends were classified as moderate risk. Studies employing interrupted time‐series analysis, difference‐in‐difference analysis or comparison with a control (e.g. a state/province without legalization) were classified as low risk. The detailed outcomes of the assessment tool are summarized in Supporting information, Table S1.

## RESULTS

**FIGURE 1:**
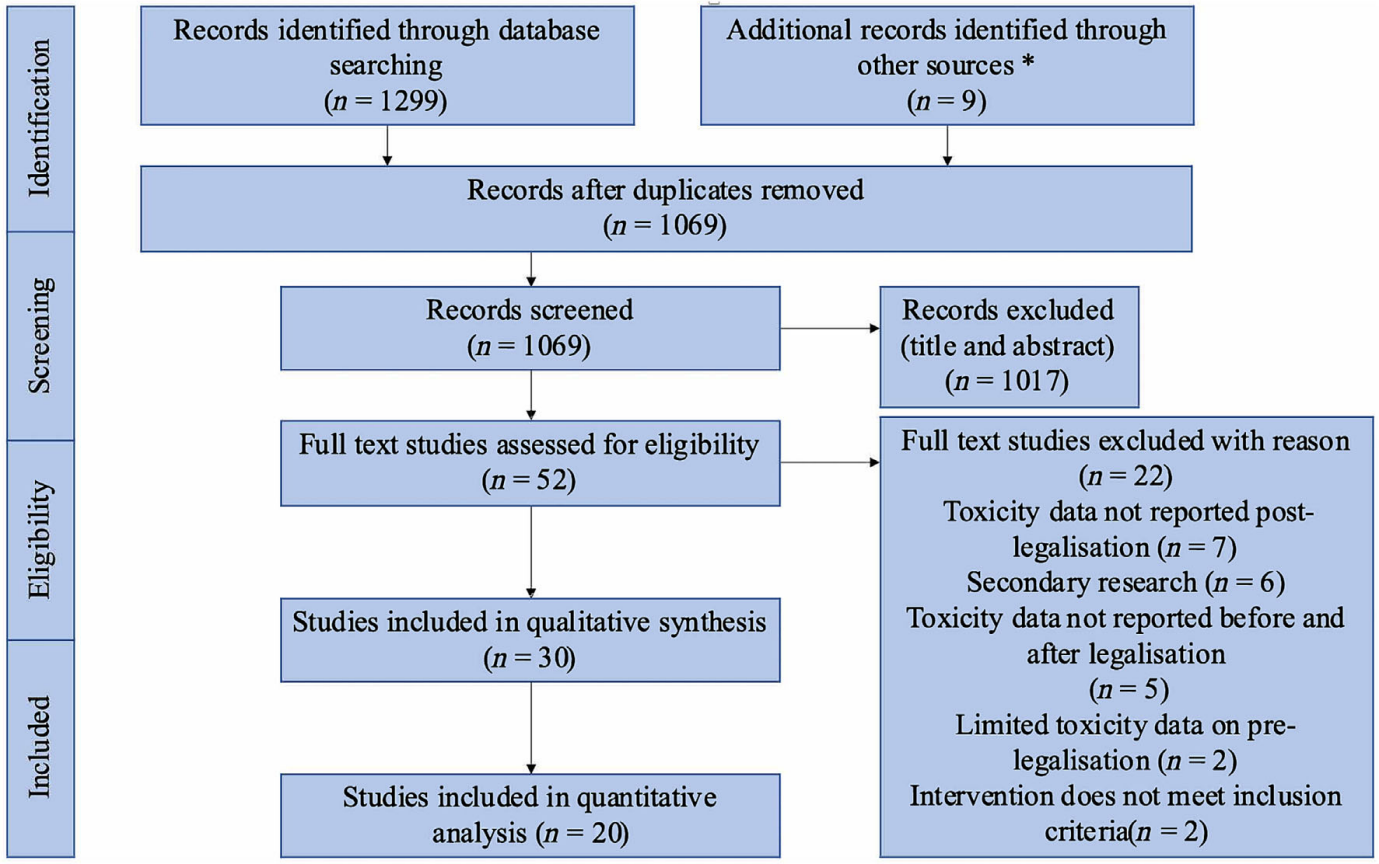
Preferred Reporting Items for Systematic reviews and Meta‐Analyses (PRISMA) diagram of study selection. *Additional sources include grey literature and citation chaining.

After database searches, 1299 studies were retrieved and 239 duplicates were removed. Nine additional studies were identified through citation chaining and hand‐searching conference abstracts. Title and abstract screening was performed on 1065 studies; 1017 irrelevant studies were excluded. Of the 52 studies reviewed by the full text, 30 met the inclusion criteria [16, 17, 20, 22, 23, 24, 25, 26, 27, 28, 29, 30, 31, 32, 33, 34, 35, 36, 37, 38, 39, 40, 41, 42, 43, 44, 45, 46, 47, 48]. Of these studies, 10 were conference abstracts [24, 25, 28, 31, 35, 36, 37, 43, 44, 45]. Twenty studies had sufficient data for quantitative synthesis [see Preferred Reporting Items for Systematic reviews and Meta‐Analyses (PRISMA) flow‐chart, Figure 1].

### Study characteristics

The included studies examined the effect of interventions on poisoning exposures in the US (*n* = 23) [17, 22, 23, 25, 26, 27, 28, 29, 30, 31, 32, 33, 35, 45, 46], Canada (*n* = 6) [16, 34, 36, 41, 47, 48] and Thailand (*n* = 1) [24]. No studies were found from Oceania, South America, Africa or Europe. Interventions included recreational cannabis decriminalization/legalization (*n* = 15) [16, 20, 32, 33, 34, 35, 36, 37, 38, 39, 40, 41, 43, 47, 48], medicinal cannabis legalization (*n* = 7) [17, 22, 23, 24, 25, 26, 27], medicinal and recreational cannabis legalization (*n* = 6) [29, 30, 31, 42, 45, 46] and CBD down‐scheduling from US Schedule I to Schedule V (*n* = 1) [28]. One study did not specify type of legalization [44]. The details of the included studies are listed in Table 1.

**TABLE 1:** Main characteristics of included studies.

Author, year [reference]	Location	Population	Year and type of intervention	Study period	Duration of post‐intervention period	Trends in cannabinoids poisoning (reported over the study period)	Data source	How was time‐varying confounding addressed?
Medicinal cannabis legalization								
Leubitz, 2021 [22]	US	Children < 6 years	Legalization of medicinal marijuana in US states (various years)	01/01/2000– 31/07/2017	NA (various states)	No significant trend in the annual mean exposures, 2000 to 2008; Increase in the annual mean of paediatric marijuana ingestions from 2008 to 2017: 81 paediatric marijuana ingestions/year (95% CI = 80.64–81.36) from 2000 to 2008; 228 ingestions/year from 2009 to 2017. Exposures more common in states with legalized marijuana	US National Poison Data System	Comparison of trends between legal/non‐legal states
Onders, 2016 [23]	US	Children < 6 years	Legalization of medicinal marijuana in US states (various years)	2000–13	Various states, up to 13 years of post‐intervention data	No significant change in marijuana exposures between 2000 and 2006: (0.6% per year; 95% CI = −2.0 to 3.3%); followed by a significant increase from 2006 to 2013: (15.0% per year; 95% CI = 12.2% to 17.8%). Following legalization, the exposure rate was 2.25 times higher (95% CI = 1.45–3.51) (in transitional states where legal status changed during the study period)	US National Poison Data System	Comparison of trends between legal/non‐legal states
Srisuma, 2020 [24]	Thailand	All ages	2019: legalization of medicinal marijuana in Thailand	01/2018–05/2019	4 months	Increase in the number of marijuana intoxication cases: 2 cases in 01/2018; 15 cases in 01/2019	Ramathibodi Poison Centre calls	NA – simple pre–post design
Wang, 2012 [25] Wang, 2013 [17]	US	Children < 12 years	2009: federal policy change that increased availability of medicinal marijuana	01/01/2005–31/12/2011	27 months (Wang 2013)	Increase in the marijuana exposures: 0 medical marijuana exposures from 01/01/2005 to 30/09/2009; 14 medical marijuana exposures from 01/10/2009 to 31/12/2011 [19]; 0 ED visits for cannabis ingestion from 01/2005 to 10/2009; 14 ED visits for cannabis ingestion 10/2009 to 12/2011 [18].	Children’s hospital/ED visits in Colorado	NA – simple pre–post design
Wang, 2014 [26]	US	Children (0–9 years)	Legalization of medicinal marijuana in US states (various years)	01/01/2005–31/12/2011	NA (various states)	Comparison between states that legalized marijuana before 2005 (‘decriminalized states’), and 2005–11 (‘transitional states’). Calls about paediatric cannabis exposures were increasing by 30.3%/year (95% CI = 22.5%–38.5%) in decriminalized states; 11.5% calls/year (95% CI = 0.4%–24.7%) in transitional states	US poison centre calls	Comparison of trends between legal/non‐legal states
Whitehill, 2019 [27]	US	Children and teenagers (0–19 years)	2012: legalization of medicinal marijuana in Massachusetts	01/01/2009–31/12/2016	4 years	Increase in paediatric cannabis exposure reported to regional poison centre: 29 cannabis exposure cases before the legalization; 69 cannabis exposure cases after the legalization	Massachusetts and Rhode Island regional Centre for Poison Control and Prevention	NA – simple pre–post design
Cannabidiol down‐scheduling								
Hinojosa, 2019 [28]	US	All ages	2018: down‐scheduling cannabidiol from US Schedule I to Schedule V	2000–18	1 year	Increase in number of cases of cannabidiol exposure: 0 cannabidiol exposure before 2015; 2 in 2015, 5 in 2016, 6 in 2017, 38 in 2018	US poison centre calls	NA – simple pre–post design
Recreational cannabis legalization								
Baraniecki, 2021 [34]	Canada	Adults	October 2018: legalization of recreational use of cannabis in Canada	17/04/2018–17/04/2019	6 months	No significant change in overall ED visits for acute cannabis toxicity. There was a significant increase (56%) in visits for young adults (18–29 years)	ED visits	Simple pre–post design
Chary, 2021 [35]	US	All ages	December 2016: legalization of recreational marijuana in Massachusetts	29/08/2012– 12/10/2019	~3 years	Increase in the number of cannabis exposures reported to the Massachusetts and Rhode Island poison centre: 120 calls/year before the legalization; 352 calls/year after the legalization.	Massachusetts and Rhode Island regional poison centre calls	N/A – simple pre–post design
Cohen, 2022 [16]	Canada	Children (0–18 years)	October 2018: legalization of recreational use of cannabis in Canada	01/01/2008–31/12/2019	14 months	No significant change in the median monthly number of paediatric cannabis exposures between pre‐legalization and peri‐post‐legalization: 2.1 per month (IQR = 1.9–2.5) versus 1.7 (IQR = 1.0–3.0). ICU admissions increased from 4.7% of cases in the pre‐legalization period to 13.6% of cases in the peri‐post‐legalization period (*P* = 0.01)	ED visits	Simple pre–post design with quantification of overall yearly trend
Coret, 2021 [36]	Canada	Children < 13 years	2018: legalization of recreational use of cannabis in Canada	03/2013–09/2020	2 years	Increase in the rate of accidental cannabis ingestion, especially after the legislation; 82% of the ED visits included in the study occurred post‐legalization	Canadian children’s hospital/ED visits	NA – simple pre–post design
Dean, 2020 [37]	US	All ages	2018: legalization of recreational use of cannabis in Michigan	2014–19	1 year	Increasing poison centre calls due to marijuana exposure: 116 cases in 2014; 348 cases in 2018. Pre‐intervention trend was already increasing	Michigan poison centre calls	Simple pre–post analysis, with description of pre‐intervention trend
Delling, 2019 [32]	US	All ages	2012: legalization passed to allow recreational cannabis in Colorado, 2014: legal purchase in retail stores	2010–14	2 years	Increase in cannabis abuse hospitalizations in Colorado in comparison to New York, RR = 1.27 (95% CI = 1.26–1.28) and Oklahoma, RR = 1.16 (95% CI = 1.15–1.17)	Health‐care cost and utilization project database in Colorado, New York and Oklahoma/cannabis abuse hospitalizations	Repeat cross‐sectional comparison with two control states
Delva‐Clark, 2020 [43]	US	Children (< 6 years)	2014: legalization of recreational marijuana sales in Colorado	01/01/2010–31/12/2019	6 years	Increase in marijuana exposure cases: 16 marijuana exposure cases in 2010; 149 in 2019	Rocky Mountain poison centre calls	Pre–post study with some quantification of pre‐trends
Myran, 2022 [47]	Canada	Children 0–9 years	October 2018: legalization of recreational use of cannabis in Canada (flower products). October 2019: edibles legalized in Ontario (did not become widely available until January 2020)	01/01/2016–31/03/2021	21 months	Monthly visits pre‐legalization: 81, post‐flower product legalization: 124, post edibles legalization: 317 Annualized rate per 100 000 population: pre‐legalization: 1.96, post‐flower product legalization: 6.14, post‐edible legalization 17.75	ED visits in Ontario	Comparison with trends from a control province where edibles were not able to be purchased
Myran, 2022a [48]	Canada	Children 0–9 years	October 2018: legalization of recreational use of cannabis in Canada (flower products). October 2019: edibles legalized in some provinces (and became available in Jan 2020): Ontario, Alberta and British Columbia	01/01/2015–30/09/2021	29 months	There were 0.95 hospitalizations per 100 000 persons/year pre‐legalization, 2.47/100000/year post‐legalization and 6.01/100000/year post‐edible legalization. Using before legalization as the reference period, the incidence rate ratio (IRR) in provinces where edibles were legal was 7.49 (95% CI = 5.92–9.48) compared to control province (Quebec): IRR = 3.04 (95% CI = 1.92–4.81)	Hospital admissions in Ontario, Alberta, British Columbia and Quebec	Calculation of incidence rate ratios with adjustment for monthly trends
Shi, 2020 [33]	US	All ages	Legalization of recreational cannabis in eight US states and the District of Columbia; commercialization of recreational cannabis in five states (various dates)	01/01/2010–31/12/2017	NA (various states)	Overall, recreational cannabis legalization was not associated with an increase in exposures. However, there was an increase in unintentional exposures: estimated percentage change between 55 and 61%, depending on modelling Recreational cannabis commercialization was analyzed separately, and was associated with an increase in exposures: estimated to be an increase between 67 to 77%.	US National Poison Data System Only data cannabis dry plants were included (i.e. no concentrates, edibles)	Difference‐in‐difference design
Thomas, 2019 [39]	US	Children (0–9 years)	2012: decriminalization of recreational marijuana in Washington; 2014: opening of recreational marijuana shops in Washington (legalization/commercialization)	01/01/2010–31/07/2016	2 years post‐legalization of retail sale	Increase in the number of marijuana exposure calls reported to the poison centre per month after shops opened: 5 marijuana exposure cases reported in 2010; 21 marijuana exposure cases reported in 2013; 48 marijuana exposure cases reported in 2015	Poison centre calls obtained from toxiCALL database	NA – simple pre–post design
Thomas, 2021 [38]	US	Children (0–9 years)	2014: opening of recreational marijuana shops in Washington (legalization/commercialization)	08/10/2007–31/10/2016	27 months	Increase in the number of unintentional marijuana exposure cases: 8 cases/6.75 years pre‐legalization; 9 cases/2.32 years post‐legalization	Children’s hospital/hospitalizations	NA – simple pre–post design
Wang, 2019 [40] Wang, 2016 [20]	US	Children (< 10 years)	2014: legalization of recreational marijuana in Colorado	1/1/2009–31/12/2017	4 years	Increase in the number of cannabis exposure cases reported to regional poison centre and children’s hospital visits: 9 cases reported to regional poison centre in 2009, 67 cases reported in 2017; 1 hospital visit regarding cannabis exposure in 2009, 36 in 2017	Colorado Poison centre calls; children’s hospital visits	Pre–post with comparison to other US states (Wang 2016)
Yeung, 2021 [41]	Canada	Children (0–17 years)	October 2018: legalization of recreational use of cannabis in Canada	01/10/2013–01/03/2020	16 months	1920 cases reported pre‐legalization (5 years, 2013–18); 602 cases reported post‐legalization (1 year 4 months October 2018–February 2020). When adjusted for population, no difference overall Subgroup analysis: unintentional ingestions increased in children 0–11 years (51 cases pre versus 40 cases post), IRR = 1.77 (95% CI = 1.42–2.20) and older adolescents 15–17 years (1534 cases pre, 439 cases post), IRR = 1.36 (95% CI = 1.07–1.71)	Edmonton and Calgary area hospitals, including St Albert and Sherwood Park/ED visits and urgent care centred data	Interrupted time‐series analysis
Medicinal and recreational cannabis legalization								
Bennett, 2021 [29]	US	Children < 6 years	Sixteen US states had legislated medicinal cannabis during or before the study period and five states and the district of Columbia had legislated recreational cannabis during the study period. There were 27 states included in the study	01/01/2004 to 12/31/2018	Varies – multiple states studied	13‐fold increase in marijuana‐related hospital encounters in young children, 2004–18. When the states with legalization were compared to those without, legalization was not found to be associated with increased rates. IRR = 1.26 (95% CI = 0.85–1.87, *P* = 0.24) for recreational marijuana; IRR = 1.23 (95% CI = 0.83–1.82, *P* = 0.30) for medicinal marijuana	The Paediatric Health Information System data/observation unit, ED and inpatient records/52 children’s hospitals	Pre–post and difference‐in‐difference model, comparing legalization status of the state
Beauchamp, 2018 [31]	US	All ages	Mixed interventions: legislation of medicinal marijuana and legalization of recreational marijuana in US states and district of Colombia	01/01/2008 to 28/02/2018	Varies – multiple states studied	An increase in exposures following legalization, with multivariate modelling indicating that recreational legalization has a stronger impact than medical legalization. 6‐fold increase in exposures following recreational law enactment. Limited numerical data available (conference abstract)	US Poison centre calls	Pre–post with some description of pre‐intervention trends
Dewey, 2020 [45]	US	Children (< 6 years)	2016: introduction of medicinal cannabis in Pennsylvania; 2011: introduction of medicinal cannabis in Delaware; 2015: decriminalization of recreational cannabis in Delaware	07/2014 to 06/2019	Varies – multiple states studied	Increase in annual marijuana exposure: 500% increase in marijuana exposure cases from 2015 to 2019.	Pennsylvania poison centre calls	NA, simple pre–post design
Wang, 2020 [30] Wang, 2017 [46]	US	All ages	2010: law change increasing availability of medicinal marijuana in Colorado; 2014: legalization of recreational marijuana in Colorado	01/01/2000 to 31/12/2018 (PCC) Hospitalization data (Wang, 2017): 01/01/2000–30/09/2015, and ED 01/01/2011–30/09/2015	5 years post‐recreational legalization (PCC data)	Increase in the number of cannabis exposure cases reported to regional poisons centre by 11.2 cases/year from 2000 to 2018. Increases seen in 2010 and 2014 following legalization. Following 2014 increase, exposures stabilized, 2014–18, followed by a 19.4% increase in cannabis exposures in 2018 in comparison to 2017 (*P* = 0.05) Hospitalization rates increased from 274 to 593/100 000 hospitalizations, 2000 to 2015	Colorado regional poison centre calls Colorado Hospital Association hospitalizations and ED visits with marijuana‐related billing codes (Wang, 2017).	Pre–post comparison with quantification of yearly trend pre‐ and post‐intervention (Wang 2020)
Wang, 2018 [42]	US	Adolescents (13–21 years)	2009: opening of medicinal marijuana sales in Colorado; 2014: implementation of recreational marijuana legalization	01/01/2005 to 31/12/2015	7 years post‐medicinal marijuana, 2 years post‐recreational	Increase in marijuana‐related ED and urgent care visits: 161 ED/urgent care visits in 2009; 777 ED/urgent care visits in 2015	Children’s hospital/ED and urgent care visits	NA, simple pre–post design
Unspecified intervention								
Spyres, 2015 [44]	US	All ages	Unspecified – various legalization interventions in the US	01/01/2010 to 31/03/2015	Varies – multiple states included in registry	Increase in the annual marijuana exposure from 2010 to 2014: 9 cases in 2010; 42 cases in 2014	ToxIC registry/poison centre calls	NA, simple pre–post design

Abbreviations: NA, not available; CI, confidence interval; ED, emergency department; IQR, interquartile range; PCC, poison control center; RR, relative risk.

Most studies investigated poisonings and exposures in children and adolescents (*n* = 19) [16, 17, 20, 22, 23, 25, 26, 27, 29, 36, 38, 39, 40, 41, 42, 43, 45, 47, 48]. More than half of the studies obtained data from poison centre calls (*n* = 18) [20, 22, 23, 24, 26, 27, 28, 30, 31, 33, 35, 37, 39, 40, 43, 44, 45, 46] and 15 studies [16, 17, 20, 25, 29, 32, 34, 36, 38, 40, 41, 42, 46, 47, 48] used hospital admissions/emergency department data (some used both data source types). The post‐intervention time‐period examined in these studies ranged from 4 months [24] to 7 years [42].

### Medicinal cannabis legalization

Medical cannabis legalization was investigated in 13 studies from the US (including Massachusetts, Colorado, Pennsylvania and Delaware) and one from Thailand [17, 22, 23, 24, 25, 26, 27, 28, 29, 30, 31, 42, 45, 46]. Most reported an increase in cannabis exposures or intoxications following legalization [17, 22, 23, 25, 26, 27, 30, 42, 45, 46]. CBD down‐scheduling was associated with increased poisons centre calls for CBD exposure [28].

### Recreational cannabis legalization and decriminalization

Twenty‐one studies examined recreational cannabis legalization/decriminalization in Canada and the US (including Colorado, Michigan, Washington, Massachusetts, Delaware and District of Colombia) [16, 20, 29, 30, 31, 32, 33, 34, 35, 36, 37, 38, 39, 40, 41, 42, 43, 45, 46, 47, 48]. Four studies were negative overall, but reported increases in poisoning in subgroup analysis [16, 33, 34, 41]. Eleven studies reported an increase in poison centre calls due to cannabis exposure [20, 30, 31, 33, 35, 37, 39, 40, 43, 45, 46]. Fourteen studies reported an increase in emergency department or hospital admissions for unintentional cannabis exposure in paediatrics, adolescents or young adults [16, 20, 25, 29, 30, 32, 34, 36, 38, 40, 41, 42, 46, 47, 48].

Canadian recreational cannabis legalization has occurred in several stages and legalization of edibles has differed by province. Sale of dried cannabis flower and oils for non‐medical use has been legal since October 2018, implemented by all provinces. Edible products (e.g. gummies) were legalized in October 2019 but each province could choose whether they approved sales of edibles. Alberta, British Columbia and Ontario approved sales of edibles in January 2020 [16, 34, 41]. We found three studies from Canada reporting on the legalization of flower‐based products and oils [16, 34, 41]. Studies focusing on the 2018 legislation found no statistically significant increase in overall cannabis‐related poisoning, but reported some increases for young adults [34] and paediatric exposures [41]. However, Cohen *et al*. found increased severity of symptoms, with higher rates of critical care unit admission, respiratory involvement and altered mental status after 2018 legalization [16].

Canadian studies on legislation affecting edibles showed different results. A repeat cross‐sectional study comparing both phases of legalization in Ontario, Canada found a significant increase in paediatric ED visits and hospitalizations, particularly following the introduction of edibles [47]. In addition, a recent study compared Canadian provinces, where edibles are legal, to a control province which allows recreational marijuana but not edibles (Quebec). Provinces where edibles are legal had much higher rates of hospital admissions for paediatric cannabis exposures compared to the control province [48].

Bennet *et al*. also compared states by legalization status in the US and found no differences when comparing states with legalized recreational marijuana to states with legalized medicinal marijuana as well as states without legalization [29]. However, there was a 13‐fold increase in total marijuana‐related hospital presentations in children from 2004 to 2018 in the 27 states analyzed [29].

Only two studies specifically examined the effect of decriminalization of recreational cannabis (as opposed to legalization) [39, 45]. Thomas *et al*. reported the effect of decriminalization of recreational cannabis in Washington in 2012 and the subsequent legalization and opening of dispensaries in 2014. This study reported an increase in paediatric exposures to marijuana following both events [39]. Dewey *et al*. examined the effect of medicinal cannabis legalization (2016) in Pennsylvania and the decriminalization of recreational cannabis (2015) in Delaware. However, Delaware cases were not reported separately, so differences between these interventions cannot be specifically evaluated [45].

Shi & Liang examined recreational cannabis legalization and commercialization separately, using a quasi‐experimental design with control states [33]. Following legalization, they did not observe a significant increase when all exposures were included; however, there was a significant increase in unintentional exposures [33]. Recreational cannabis commercialization resulted in a significant and consistent increase in poisons centre exposures [33]. This study included dried plant cannabis only (i.e. no edibles or concentrates), which may account for the lack of increase observed overall poisonings [33]. Indeed, other studies have indicated that increases in recent years are not being driven by smokeable marijuana [30].

### Quantitative synthesis

**FIGURE 2:**
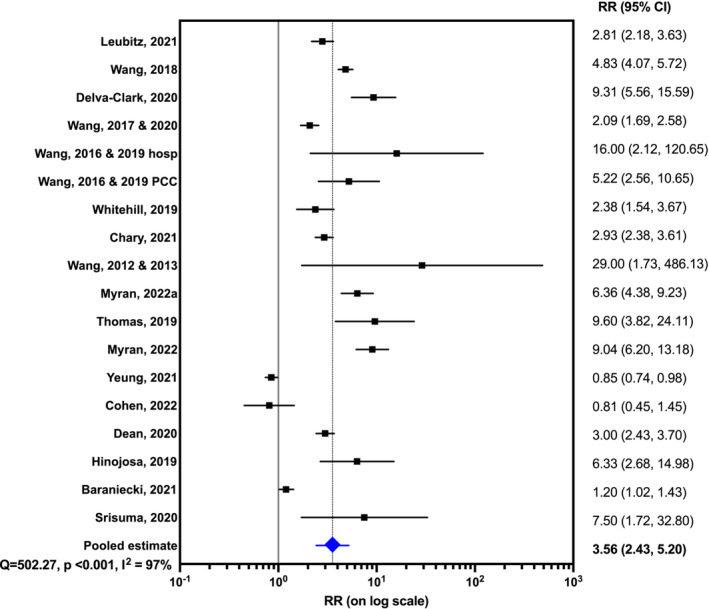
Forest plot displaying the relative risk (RR) of cannabis poisoning following the intervention from all studies where raw numbers were available for quantitative synthesis. A random effects model was used. RRs have been plotted on a logarithmic (base 10) scale. Studies have been ordered by duration of time between the main intervention and the final data point (shortest on the bottom).

Twenty studies were suitable for quantitative synthesis, six studies of all ages [24, 28, 30, 35, 37, 46], one of adults [34] and the remainder focusing on the paediatric population [16, 17, 20, 22, 25, 27, 39, 40, 41, 42, 43, 47, 48]. Due to partial data overlap results from the following studies were pooled: Wang 2017 [46] and Wang 2020 [30]; Wang 2016 [20] and Wang 2019 [40]; and Wang 2012 [25] and Wang 2013 [17]. One study used data from Thailand, five used Canadian data and the remainder were from the US. The rates of cannabis poisoning generally increased after interventions with a pooled RR estimate of 3.56 (95% CI = 2.43–5.20, random‐effects model, Figure 2). However, results from legalizing/decriminalizing medicinal or recreational cannabis were highly heterogeneous (*I* ^2^ = 97%). The RR varied greatly, from 0.81 to 29.00 [16, 17]. There was one negative study, one where the 95% CI crossed one, the remainder had RR above one, and 95% CI not crossing one. We conducted several subgroup analyses, and RR increased after the interventions for all the study subgroups we explored (Table 2). RR appeared to be lower for studies that only used hospital data (RR = 3.42, 95% CI = 1.60–7.31) and studies that included all ages (RR = 2.63, 95% CI = 1.78–3.88), and higher for those focused on paediatrics (RR = 4.31, 95% CI = 2.30–8.07). Studies that focused on the medical use of cannabis reported a higher RR and were less heterogeneous (RR = 3.67, 95% CI = 2.36–5.71, *I* ^2^ = 72%) than those that included the recreational use of cannabis (RR = 3.49, 95% CI = 2.22–5.48, *I* ^2^ = 97%).

**TABLE 2:** Relative risk (RR) of cannabis poisoning following legalization by subgroups.

Subgroup analysis	Studies (*n*)	RR of cannabis poisoning (95% CI)	*I* ^2^ value
Data source			
Poison centre	10	3.61 (2.74–4.74)	84%
Hospital	8	3.42 (1.60–7.31)	98%
Age group			
All ages	6	2.63 (1.78–3.88)	93%
Paediatric	12	4.31 (2.30–8.07)	97%
Type of legalization			
Medicinal use	4	3.67 (2.36–5.71)	72%
Recreational use	14	3.49 (2.22–5.48)	97%
Study type			
Grey literature	4	3.46 (2.30–5.21)	84%
Peer‐reviewed publications	14	3.56 (2.19–5.80)	97%

Abbreviation: CI, confidence interval.

### Clinical effects, outcomes and disposition

**FIGURE 3:**
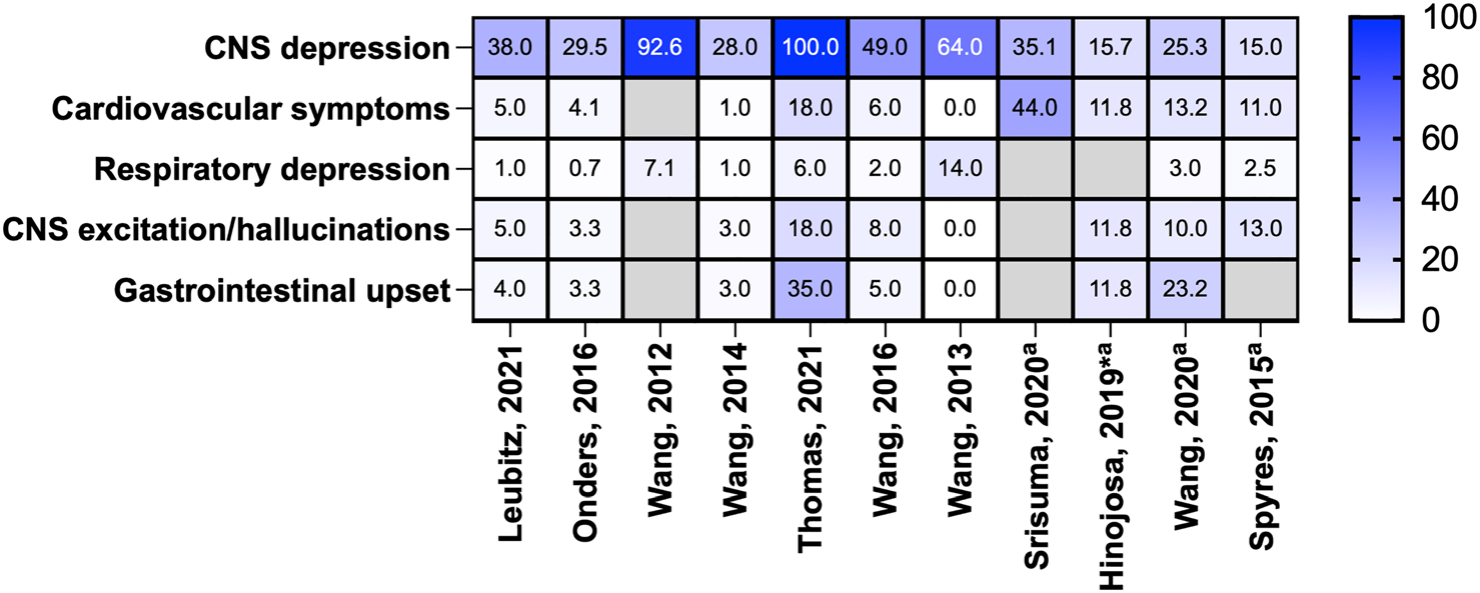
Heat‐map displaying the percentages of clinical effects of cannabis exposures. Unless otherwise indicated, studies were of the paediatric population. Cells shaded grey indicate the study did not report that parameter. ^a^Study focused on all ages; *study only examined cannabidiol exposures.

**FIGURE 4:**
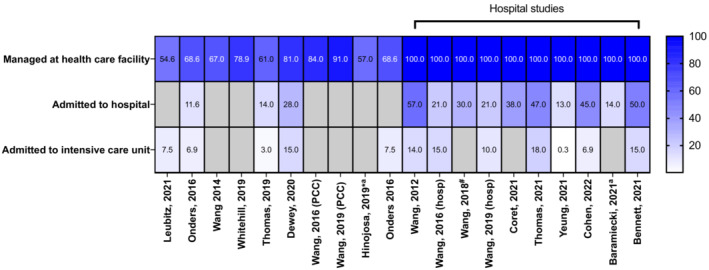
Heat‐map displaying the percentages of people managed at a health‐care facility, admitted, and admitted to critical care/intensive care. Studies only reporting on hospitalized patients are indicated with the square bracket. Unless otherwise indicated, studies were of the paediatric population. Cells shaded grey indicate the study did not report that parameter. ^a^Study focused on all ages; ^#^study of adolescents only; *study only examined cannabidiol exposures.

**FIGURE 5:**
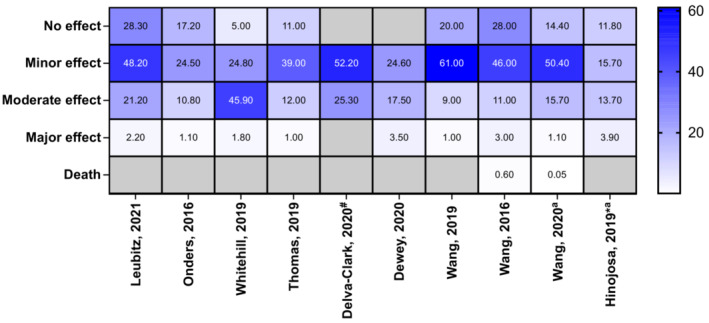
Heat‐map showing clinical outcomes categories (in percentages) reported by poisons centre studies. Unless otherwise indicated, studies were of the paediatric population. Cells shaded grey indicate that the study did not report that parameter. ^a^Study focused on all ages; ^#^study of adolescents only; *study only examined cannabidiol exposures. Wang 2016 and Wang 2020 reported the same death.

Reported clinical effects of the poisonings were described by many studies, including lethargy, drowsiness, dizziness, hypertension, palpitations, tachycardia, nausea, vomiting, irritability, agitation, coma and CNS depression (Supporting information, Table S2). CNS depression was most commonly reported in children (Figure 3). There was a total of 72 reports of coma from all studies; however, there may be some overlap between data sources used. The majority of paediatric exposures were managed in a health‐care facility (range = 55–100%), intensive care unit (ICU) admissions were also common (Figure 4). ‘Minor effect’ and ‘moderate effect’ were the most frequently reported outcomes in children (Figure 5). Studies that reported on all age groups reported more patients as asymptomatic (‘no effect’) than those focusing on children. One death was reported after legalization of recreational marijuana in Colorado (Wang 2016 and Wang 2020 appear to report the same paediatric case, Supporting information, Table S2) [20, 30].

### Quality assessment

We assessed the quality of each study using the ROBINS‐I tool (Supporting information, Table S1). Most studies received a score of low to medium risk of bias. Main sources of bias were not accounting for time‐varying confounders (also displayed in Table 1), missing data, lack of detail about the intervention and imprecision in the intervention time (e.g. in the US where legislation varies across states and time). Almost half (*n* = 14) the studies simply presented pre‐ and post‐intervention counts/rates, without accounting for broader trends [17, 24, 25, 27, 28, 34, 35, 36, 38, 39, 40, 42, 44, 45], and some presented some quantification of pre‐intervention trends [16, 30, 31, 37, 43]. Only a few stronger quasi‐experimental study designs were used, including an interrupted time‐series analysis [41], a repeat cross‐sectional study with segmented regression analysis [48] and quasi‐experimental difference‐in‐differences design, with comparison to control states [29, 33]. Several studies adjusted for confounding by using control states/provinces where cannabis laws were unchanged or compared to overall national trends [20, 22, 23, 26, 32, 40, 47]. Two studies using a more robust approach were negative overall (but showed significant increases for some subgroups) [33, 41].

## DISCUSSION

This systematic review found that cannabis legislation or decriminalization was associated with an increased incidence of cannabis poisoning. The probable explanation is that the legislation increased use which also increases poisoning. The modification of cannabis laws could increase perceived acceptability of cannabis use in the community, increasing use and dependence [49]. Increased medical and non‐medical use can increase the risk of poisoning due to availability and access. Increased use and availability of edibles appears to be an important driver of the increase in poisonings, particularly in the paediatric population. Despite legalization and decriminalization in many other countries, the vast majority of evidence was from the US and Canada.

Most studies included in this review reported increases in poisoning exposures following legalization/decriminalization of either medicinal or recreational cannabis. Studies which did not report a statistically significant change for the primary outcome of overall poisoning often found increases among subgroups, e.g. children [34, 41] or ICU admissions [16]. The three studies in Canada which reported no significant change in cannabis exposure after recreational cannabis legalization were in the earlier phase of legalization, where flower products were legalized before edibles [16, 34, 41]. There was also an additional lag between legalization of edibles and their commercial availability for purchase [41]. Changes in paediatric poisonings due to edibles have probably not yet been covered in these study periods. The only study with sufficient data post‐edible availability showed a significant increase in paediatric hospital presentations and admissions [48]. More long‐term studies are needed to continue to monitor the impacts of recreational cannabis legalization in Canada, especially considering that most unintentional paediatric poisonings are due to edibles [27]. Bennet *et al*. reported similar incidence rate ratios for US states with different legalization statuses for marijuana (recreational versus medicinal versus non‐legalized) [29]. However, these comparisons may be confounded by people purchasing marijuana from neighbouring states with more liberal laws [29].

In contrast to North America, Uruguay’s recreational cannabis legalization is heavily controlled by the government. Uruguay has a mandatory registry and sets a limit to grams of cannabis used by each individual [50]. They do not allow advertising, selling to tourists or edibles [50]. The different legalization models would be expected to have a different impact on outcomes. However, we could not find any studies assessing the impact of the Uruguayan model on poisonings.

The assumption that increased poisonings are a result of increased use requires examination of the impact of legislation on overall use patterns. Cannabis use in the last year increased 70% in Canada post‐legalization [51], with a particularly large increase in use of edibles following legalization of their retail sale [52]. In the US, Cerdá and colleagues examined the impact of recreational cannabis legalization on use [53]. Adjusting for trends observed in control states, they found that cannabis use did not increase in adolescents and young adults following legalization of recreational cannabis. However, frequent use increased in adults. Similar results were observed following medicinal marijuana legalization [54]. Increasing use in older adults could increase poisoning across all age groups by increasing availability in households. A comparison of prevalent use in states where cannabis is legal versus illegal shows higher rates of use in legal states, particularly with edibles [52]. Taken together, this indicates that patterns of use are influenced by legalization, although it can be difficult to separate this effect from baseline trends. This underscores the importance of accounting for time‐varying confounding (discussed further in ‘Strengths and limitations’, below).

Cannabis exposures in children are of particular concern, as they have the potential for severe toxicity, including coma, neurological and cardiovascular effects. While exact circumstances surrounding exposures was not the focus of this review, adverse reactions or dosing errors with medicinal cannabis products used in children were rare [27]. In support of this, one paediatric study found low (< 10) counts of diagnoses of conditions for which medical cannabis is commonly used (e.g. epilepsy, cancer) [40]. Rather, exposures in young children tend to be unintentional exposures to cannabis products owned by parents, older siblings or care‐givers [27]. These findings are potentially due to the significant rise in the availability of edible products such as cookies, gummies and brownies, which are attractive to children [49, 55]. Harm minimization approaches need to be applied, including the sale of edible cannabis products in child‐resistant containers, opaque packaging and enhancing parents’ awareness of the risk of edible marijuana for children [56]. The increase in paediatric cannabis exposures may also be driven by increasing recreational use of cannabis among older children and adolescents [27].

We found that data on the impact of cannabis decriminalization are lacking, with most studies focusing on legalization. Decriminalization was often followed by legalization, and studies tended to investigate the effect of legalization together with decriminalization [39]. Publication bias is another potential reason for the limited data on decriminalization (which would have been expected to have much less of an impact on use and misuse). There may be some unpublished studies that did not report a significant change in poisoning after the decriminalization [57].

A minority of studies reported other impacts of the intervention in addition to poisoning. Data on the co‐ingestion of other substances conflicted between studies [32, 41]. One study reported an increase in alcohol intake after cannabis legislation [32]. A prior study reported that cannabis use is significantly associated with a rise in the ingestion of alcohol [58]. In contrast, one study reported a decrease in cocaine, alcohol and opioid consumption in adolescents [41]. Reduced consumption of opioids is consistent with a previous systematic review, which reported that the legalization of medical cannabis led to a decrease in prescription opioids dispensing [59]. That research found a decrease in opioid overdose mortality by 21% in states with medicinal cannabis laws, 1999–2010 [59]. However, this result was not replicated when extended to 2017, with a 23% increase in opioid mortality seen [60].

SCRAs are more dangerous than naturally occurring cannabinoids, with a different pharmacological profile including psychostimulant‐like properties [61]. These products are widely used in the community and present a law enforcement challenge [62]. Most users prefer natural cannabis to SCRAs and cite perceived legality and the fact that SCRAs are not detectable on routine drug screening as reasons for use [63, 64]. One potential impact of the legalization of recreational cannabis is a reduction in harm from SCRAs. A study has shown a reduction in SCRA exposures following medical and recreational cannabis legalization [65]. Further research is needed in this area.

### Strengths and limitations

This study had several strengths. A comprehensive search strategy was used, with no language restrictions. Grey literature conference abstracts were also included, which provided more data. Screening and data extraction was performed by two independent authors, with conflicts resolved by discussion with a third author, reducing the risk of bias in the selection of studies.

Despite these strengths, this review had some limitations. We focused on acute poisoning, and do not report on harms from longer‐term exposure such as hyperemesis syndrome, chronic psychosis and chronic cardiovascular illness. Hyperemesis syndrome can be severe and life‐threatening, and thus we may be underestimating harms from subacute toxicity by excluding this. In addition, there was often a delay between law enactment and implementation of the intervention in the community, including the opening of cannabis dispensaries [32, 42]. This lag can influence the interpretation of any changes in health outcomes or limit the findings from studies with a short post‐intervention period. Our quantitative analysis is limited by the high heterogeneity observed, which was able to be slightly reduced by subgroup analysis. We examined exposures to cannabis, as well as THC and CBD specifically. However, we found no studies that specifically evaluated different isomers (i.e. delta‐9‐THC versus the newly emerging delta‐8‐THC and delta‐10‐THC) [66].

The main quality issue of the identified studies was high risk of bias due to time‐varying confounding. Many studies simply presented pre‐ and post‐intervention counts/rates, rather than using more robust quasi‐experimental designs. Increases observed by those studies could be simply a result of longer‐term trends. This limitation also applies to our quantitative synthesis. Other factors, besides legalization, could influence trends in adult cannabis use, which are not accounted for with a simple pre–post study design. This includes changing community attitudes to cannabis use (reductions in perceptions of risk since 2000) [67] and the increasing potency of the illicit cannabis supply [68]. However, the fact that increases seem to be more influenced by the availability of edible forms of cannabis supports the conclusion that the potency of flower/plant products is not a major driver of the increase. Future studies should employ robust methods, including difference‐in‐difference analysis, interrupted time‐series analysis and/or use of control jurisdictions to increase confidence in findings.

## CONCLUSION

Medicinal or recreational cannabis legalization is associated with increased cannabis poisoning in adults, adolescents and children. However, there was much unexplained heterogeneity in the outcomes measured. The majority of evidence is from North America, although medicinal cannabis is legal in many other countries, and recreational cannabis in some countries [2, 3, 4, 5, 6, 7, 8]. It is important that public health agencies consider applying harm minimization approaches to limit the impact of cannabis legislation on acute poisonings, especially as legalization or recreational cannabis continues to be debated. With changing attitudes and perception of risk, there is a need for greater public awareness of the risks of cannabis poisoning, particularly to young people.

## AUTHOR CONTRIBUTIONS

**Sara Allaf:** Data curation (lead); formal analysis (lead); writing—original draft (lead). **Jessy Sheiway Lim:** Data curation (supporting); formal analysis (supporting); writing—review and editing (supporting). **Nicholas A Buckley:** Conceptualization (equal); formal analysis (supporting); methodology (equal); supervision (supporting); visualization (equal); writing—review and editing (supporting). **Rose Cairns:** Conceptualization (equal); formal analysis (supporting); writing—review and editing (lead); supervision (lead); methodology (equal); visualization (equal).

## DECLARATION OF INTERESTS

None to declare.

## Supporting information


**Table S1.** ROBINS‐I Risk of bias quality assessment [21].
**Table S2.** Clinical effects and clinical outcomes of cannabis poisoning reported in the studies.

## Data Availability

These data were derived from the referenced studies available in the public domain.
