# Supplementary material for: The impact of cannabis legalization and decriminalization on acute poisoning: A systematic review
Source: Addiction. 2023 Jul 26;118(12):2252–74. doi: 10.1111/add.16280 (PMC10952774; doi:10.1111/add.16280)
Supplement: Supplementary file 1 — Table S1. ROBINS‐I Risk of bias quality assessment [21]. Table S2. Clinical effects and clinical outcomes of cannabis poisoning reported in the studies. [file ADD-118-2252-s001.docx]

**The Impact of Cannabis Legalisation and Decriminalisation on Acute Poisoning: A Systematic Review**

**SUPPLEMENTARY MATERIAL:**

**Supplementary methods:**

*Search Strategies*

**Medline (Ovid)**

1. (Decriminalis* or decriminaliz*).mp. (879)
2. Legislation, Drug/ (10589)
3. Health Policy/ (70633)
4. (Health policy or health policies).mp. (97244)
5. (Post legalis* or post lealiz*).mp. (7)
6. (legalis* or legaliz*).mp. (4979)
7. Jurisprudence/ (29957)
8. Jurisprudence.mp. (287420)
9. Drug Approval/ (16262)
10. Drug approval.mp. (17171)
11. Legal aspect*.mp. (5549)
12. 1 or 2 or 3 or 4 or 5 or 6 or 7 or 8 or 9 or 10 or 11 (393754)
13. Cannabinoid*.mp. (26265)
14. Cannabidiol.mp. (4557)
15. exp Cannabinoids/ (16385)
16. Cannabis.mp. (27606)
17. Cannabis/ (11421)
18. Mari?uana.mp. (24090)
19. Marinol.mp. (92)
20. Nabilone.mp. (375)
21. Tetrahydrocannab*.mp. (8525)
22. THC.mp. (8549)
23. Cesamet.mp. (23)
24. Sativex.mp. (217)
25. Epidiolex.mp. (125)
26. Dexanabinol.mp. (51)
27. 13 or 14 or 15 or 16 or 17 or 18 or 19 or 20 or 21 or 22 or 23 or 24 or 25 or 26 (62459)
28. Poisoning/ (22730)
29. Poisoning*.mp. (150251)
30. Toxicity.mp. (775918)
31. (Drug-Related Side Effects and Adverse Reactions).mp. (35998)
32. (Adverse drug event* or adverse drug reaction* or drug side effect* or drug toxicity).mp. (35486)
33. Drug Overdose/ (13089)
34. Overdos*.mp. (30938)
35. Drug intoxica*.mp. (984)
36. Intoxication.mp. (51540)
37. Toxic ingestion*.mp. (231)
38. Adverse outcom*.mp. (35362)
39. 28 or 29 or 30 or 31 or 32 or 33 or 34 or 35 or 36 or 37 or 38 (1027356)
40. (Lavi et al., 2016)12 and 27 and 39

March 2022: 305

**Embase (Ovid)**

1. (decriminaliz* or decriminalis*).mp. (1121)
2. Drug legislation.mp. (15063)
3. health care policy/ (208414)
4. (Health policy or health policies).mp. (43861)
5. (Post legalis* or post lealiz*).mp. (8)
6. (legalis* or legaliz*).mp. (5954)
7. jurisprudence/ (24820)
8. jurisprudence.mp. (48351)
9. 1 or 2 or 3 or 4 or 5 or 6 or 7 or 8 (290931)
10. exp cannabinoid/ (77440)
11. cannabinoid*.mp. (37814)
12. cannabis.mp. (59211)
13. cannabidiol.mp. (7843)
14. mari?uana.mp. (21697)
15. marinol.mp. (627)
16. Nabilone.mp. (1546)
17. nabilone/ (1482)
18. Cesamet.mp. (301)
19. Sativex.mp. (778)
20. Epidiolex.mp. (336)
21. dexanabinol.mp. (1255)
22. dexanabinol/ (1247)
23. 10 or 11 or 12 or 13 or 14 or 15 or 16 or 17 or 18 or 19 or 20 or 21 or 22 (101285)
24. poisoning*.mp. (115284)
25. toxicity.mp. (1255688)
26. (Adverse drug event* or adverse drug reaction* or drug side effect* or drug toxicity).mp. (2154595)
27. adverse drug reaction/ (266616)
28. exp drug overdose/ (32306)
29. exp drug intoxication/ (22592)
30. overdos*.mp. (54997)
31. drug intoxica*.mp. (23187)
32. intoxication.mp. (261208)
33. toxicity/ or "toxicity and intoxication"/ (152693)
34. 24 or 25 or 26 or 27 or 28 or 29 or 30 or 31 or 32 or 33 (2757689)
35. 9 and 23 and 34

March 2022: 891

**EBM Reviews - Cochrane Central Register of Controlled Trials**

1. (Decriminalis* or decriminaliz*).mp. (8)
2. Legislation, Drug/ (10)
3. Health Policy/ (207)
4. (Health policy or health policies).mp. (1087)
5. (Legalis* or legaliz*).mp. (100)
6. Jurisprudence/ (30)
7. Jurisprudence.mp. (95)
8. Drug Approval/ (123)
9. drug approval.mp. (1175)
10. legal aspect*.mp. (236)
11. 1 or 2 or 3 or 4 or 5 or 6 or 7 or 8 or 9 or 10 (2673)
12. cannabinoid*.mp. (1253)
13. exp Cannabinoids/ (906)
14. cannabis.mp. (2954)
15. Cannabis/ (350)
16. cannabidiol.mp. (872)
17. mari?uana.mp. (2177)
18. marinol.mp. (36)
19. Nabilone.mp. (165)
20. Tetrahydrocannab*.mp. (1112)
21. Cesamet.mp. (7)
22. Sativex.mp. (160)
23. Epidiolex.mp. (109)
24. dexanabinol.mp. (9)
25. 12 or 13 or 14 or 15 or 16 or 17 or 18 or 19 or 20 or 21 or 22 or 23 or 24 (5287)
26. Poisoning/ (216)
27. poisoning*.mp. (1994)
28. toxicity.mp. (47169)
29. (Drug-Related Side Effects and Adverse Reactions).mp. (1671)
30. (Adverse drug event* or adverse drug reaction* or drug side effect* or drug toxicity).mp. (73006)
31. overdos*.mp. (2080)
32. Drug Overdose/ (180)
33. drug intoxica*.mp. (186)
34. intoxication.mp. (3491)
35. Drug overdose.mp. (1168)
36. 26 or 27 or 28 or 29 or 30 or 31 or 32 or 33 or 34 or 35 (114920)
37. 11 and 25 and 36

March 2022: 18

**Scopus**

( TITLE-ABS-KEY ( "Drug legislation" OR "Health policy" OR "health policies" OR legali?ation OR jurisprudence OR "Drug Approval" OR "legal aspect*" ) AND TITLE-ABS-KEY ( cannabinoid* OR cannabis OR mari?uana OR marinol OR nabilone OR tetrahydrocannab* OR thc OR cesamet OR sativex OR epidiolex OR dexanabinol ) AND TITLE-ABS-KEY ( poisoning* OR toxicity OR "adverse drug event*" OR "adverse drug reaction*" OR "drug side effect*" OR "drug toxicity" OR "Drug Overdose" OR overdos* OR "drug intoxica*" OR intoxication ) ) AND ( LIMIT-TO ( SUBJAREA , "PHAR" ) )

March 2022: 85

**Supplementary Table 1:** ROBINS-I Risk of bias quality assessment [21].

| **Author** | **Location** | **Year** | **Risk of bias due to confounding** | **Selection bias of participants** | **Bias in classification of intervention** | **Bias due to deviations from intended interventions** | **Bias due to missing data** | **Bias in measurement of outcome** | **Selective reporting of outcome** |
| --- | --- | --- | --- | --- | --- | --- | --- | --- | --- |
| Leubitz [22] | USA | 2021 | Low | Mid (children < 6 years) | Low | Low | Low | Low | Low |
| Onders [23] | USA | 2016 | Low | Mid (children < 6 years) | Mid-High (various legalisation interventions in the USA-difficult to identify) | Low | High (missing data) | Low | Low |
| Sirsuma [24] | Thailand | 2020 | High (simple pre-post) | Low | Low | Low | low | Low | Low |
| Wang [25] | USA | 2012 | High (simple pre-post) | Mid (Children < 12 years) | Low | Low | low | Low | Low |
| Wang [17] | USA | 2013 | High (simple pre-post) | Mid (Children < 12 years) | Low | Low | Low | Low | Low |
| Wang [26] | USA | 2014 | Low | Mid (Children (0- 9 years)) | Mid-High (various legalisation interventions in the USA-difficult to identify) | Low | High (missing data) | Low | Low |
| Whitehill [27] | USA | 2019 | High (simple pre-post) | Mid (Children and teenagers (0-19 years)) | Low | Low | Low | Low | Low |
| Hinojosa [28] | USA | 2019 | High (simple pre-post) | Low | Low | Low | Low | Low | Low |
| Shi [33] | USA | 2020 | Low | Low | Mid-High (various legalisation interventions in the USA-difficult to identify) | Low | High (missing data) | Low | Low |
| Chary [35] | USA | 2021 | High (simple pre-post) | Low | Low | Low | Low | Low | Low |
| Dean [37] | USA | 2020 | Mid (simple pre-post but mention of pre-intervention trend) | Low | Low | Low | Low | Low | Low |
| Thomas [39] | USA | 2019 | High (simple pre-post) | Mid (Children (0- 9 years) | Low | Low | Low | Low | Low |
| Wang [40] | USA | 2019 | High (simple pre-post) | Mid (Children <10) Years | Low | Low | High (missing data) | Low | Low |
| Delva-Clark [43] | USA | 2020 | Mid (pre-post with some quantification of pre-intervention trend) | Mid (Children (< 6 years) | Low | Low | Low | Low | Low |
| Coret [36] | Canada | 2021 | High (simple pre-post) | Mid (Children < 13 years) | Low | Low | High (missing data) | Low | Low |
| Thomas [38] | USA | 2021 | High (simple pre-post) | Mid (Children (0-9 years) | Low | Low | High (missing data) | Low | Low |
| Wang [20] | USA | 2016 | Low | Mid (Children (0-9 years) | Low | Low | Low | Low | Low |
| Delling [32] | USA | 2019 | Low | Low | Low | Low | High (missing data) | Low | Low |
| Yeung [41] | Canada | 2021 | Low | Mid children (0-17 years) | Low | Low | Low | Low | Low |
| Cohen [16] | Canada | 2022 | Mid (pre-post with additional analysis of correlation of year and number of events) | Mid (Paediatric (0-18 years) | Low | Low | Low | Low | Low |
| Baraniecki [34] | Canada | 2021 | High (simple pre-post analysis) | Mid (adults only) | Low | Low | Low | Low | Low |
| Wang [30] | USA | 2020 | Low | Low | Mid (Legalisation of medicinal and recreational cannabis-difficult to identify) | Low | High (missing data) | Low | Low |
| Bennett [29] | USA | 2021 | Low | Mid (Children < 6 years) | Mid-High (various legalisation interventions in the USA-difficult to identify) | Low | High (missing data) | Low | Low |
| Beauchamp [31] | USA | 2018 | Mid (pre-post with some quantification of slope) | Low | Mid-High (various legalisation interventions in the USA-difficult to identify) | Low | High (missing data) | Low | Low |
| Dewey [45] | USA | 2020 | High (simple pre-post) | Mid (children (6 years) | Mid-High (various legalisation interventions in the USA-difficult to identify) | Low | High (missing data) | Low | Low |
| Wang [42] | USA | 2018 | High (simple pre-post) | Mid (adolescents 13-21 years) | Mid (Legalisation of medicinal and recreational cannabis-difficult to identify) | Low | Low | Low | Low |
| Spyres [44] | USA | 2015 | High (simple pre-post) | Low | High (no details of intervention) | Low | High (missing data) | Low | Low |
| Myran [47] | Canada | 2022 | Low | Mid (children 0-9 years) | Low | Low | Low | Low | Low |
| Myran [48] | Canada | 2022a | Low | Mid (children 0-9 years) | Low | Low | Low | Low | Low |
| Wang [46] | USA | 2017 | Low | Low | Mid (Legalisation of medicinal and recreational cannabis-difficult to identify) | Low | Low | Low | Low |

**Supplementary Table 2:** Clinical effects and clinical outcomes of cannabis poisoning reported in the studies

| **Study** | **Clinical effects and outcome** | **Disposition/management** |
| --- | --- | --- |
| Leubitz, 2021 [22] | Drowsiness/lethargy (38%, n=1119), ataxia (6%, n=183), tachycardia (5%, n=142), vomiting (4% n=125), agitated/irritable (4%, n=123), mydriasis (4%, n=114), confusion (3%, 98), respiratory depression (1% n=38), coma (1% n=27), hallucinations (1% n=27).  No effect (28.3%n n=508), minor effect (48.2%, n=865), moderate effect (21.3%, n=383), major effect (2.2%, n=40). | Managed at a healthcare facility (54.6%), admitted to critical care (7.5%, n=224). |
| Onders, 2016 [23] | Neurological effects (46%, n=896), including: drowsiness or lethargy (29.5%), ataxia (5.4%), agitation or irritability (3.3%), confusion (2.5%). Serious effects were less common and included coma (0.9%, n=17), respiratory depression (0.7%, n=14), and single (n=6) or multiple (n=4) seizures. Cardiovascular effects (4.1%, n=80), gastrointestinal effects (3.3%, n=65).  No clinical effect (17.2%, n=339), minor effect (24.5%, n=482), moderate effect (10.8%, n=213), major effect (1.1%, n=21). | Managed at a healthcare facility (68.6%, n=1344) admitted to a noncritical care unit (11.6%, n=229), admitted to critical care (6.9%, n=136). |
| Srisuma, 2020 [24] | Coma (1.3%, n=4), palpitation (36.4%, n=110), tachycardia (44.0%, n=133), dizziness (35.1%, n=106), high blood pressure (36.4%, n=110). | Intubation (1.3%, n=4). |
| Wang, 2012 [25] | Central nervous system effect (e.g. ataxia/lethargy) (92.6%, n=13), respiratory insufficiency (7.1%, n=1). | Study population was ED presentations. ED observation only (36%, n = 5), admission (57%, n=8), critical care admission (14%, n=2). |
| Wang, 2013 [17] | Lethargy (64%, n=9), hypoxia or respiratory insufficiency (14%, n=2), ataxia (7%, n=1), dizziness (7%, n=1). | Study population was patients presenting to the ED. Patients were observed in ED (36%, n=5), admitted (57%, n=8), admitted to critical care (14%, n=2). |
| Wang, 2014 [26] | Drowsiness/lethargy (28%, n=272), ataxia (5%, n = 46), agitated/irritable (3%, n=32), confused (3%, n=27), nausea/vomiting (3%, n=30), hypotension/bradycardia and respiratory depression (1%, n=10). | Needed health care facility evaluation (67%, n=660), not managed at health care facility (30%, n=296). |
| Whitehill, 2019 [27] | Major effect (1.8%, n=4), moderate (45.9%, n=100), minor effect (24.8%, n=54), no effect (5.0%, n=11) | 78% (n=172) were in a healthcare facility at the time of call. |
| Hinojosa, 2019 [28] | Vomiting (n=6, 11.8%), drowsiness/lethargy (n=8, 15.7%),  agitated/irritable (n=6, 11.8%), tachycardia (n=7, 13.7%), hypertension (n=6, 11.8%).  No effect (11.8%, n=6), minor effect (15.7%, n=8), moderate effect (13.7%, n=7), major effect (3.9%, n=2). | In hospital/referred to hospital (57%, n=29). |
| Shi, 2020 [33] | N/A | N/A |
| Chary, 2021 [35] | N/A | N/A |
| Dean, 2020 [37] | N/A | N/A |
| Thomas, 2019 [39] | No effect (11%, n=18), minor effect (39%, n=62), moderate effect (12%, n=20), major effect (1%, n=1), the remainder unknown/not followed. | In hospital/referred to hospital (61%, n=98), critical care admission (3%, n=5), admitted to non-critical care unit (14%, n=22). |
| Wang, 2019 [40] | No effect (20%, n=23), minor effect (61%, n=71), moderate effect (9%, n=11), major effect (1%, n=1). | Hospital data: managed at inpatient setting (21%, n=11), intensive care unit (10%, n=5).  Regional poison centre data: managed or referred to a health care facility (91%, n=106), managed at home (8%, n=9). |
| Delva-Clark, 2020 [43] | Minor effect (52.2%), moderate effect (25.3%). | Not managed at a health care facility (58.2%). |
| Coret, 2021 [36] | N/A | Study population was people presenting to ED, admission (38%, n=15). |
| Thomas, 2021 [38] | Lethargy/drowsiness/altered mental status (100%, n=17), ataxia (41%, n=7), agitation (18%, n=3), hallucinations (6% n=1), respiratory depression (6%, n=1), tachycardia (18%, n=3), nausea/vomiting (35%, n=6). | Study population was hospital presentations. Discharged from emergency department (35%, n = 6), admitted (47%, n=8), critical care admission (18%, n=347). |
| Wang, 2016 [20] | Study included hospital and poisons centre data.  From poisons centre data. Agitation (8%, n=13), drowsiness/lethargy (49%, n=80), ataxia/dizziness (12%, n=20), tachycardia (6%, n=9), respiratory depression (2%, n=4), vomiting (5%, n=8), bradycardia/hypotension (2%, n=4), seizures (3%, n=5), dystonia/muscle rigidity (2%, n=4).  No effect (28%, n=45), minor effect (46%, n=75), moderate effect (11%, n=18), major effect (3%, n=4). One paediatric death (an 11 month old who died from myocarditis with detectable tetrahydrocannabinol post-mortem). | From hospital data: ED observation only (65%, n=40), admitted (21%, n=13), critical care admission (15%, n=9).  Poisons centre data: in hospital/referred to hospital (84%, n=137). |
| Delling, 2019 [32] | N/A | N/A |
| Yeung, 2021 [41] | Seizures (1%, n=19) | Study population was patients presenting to ED. Admitted (13%, n=339), admitted to critical care unit (0.3%, n=8) |
| Cohen, 2022 [16] | Altered mental status (17.4%, n=52, seizures (3%, n=9) respiratory involvement (54%, n=161). | Study population was people presenting to ED. Admitted/hospitalized (45%, n= 134), ICU (7%, n=20), discharged home (55%, n=164). |
| Baraniecki, 2021 [34] | Chief complaint: bizarre behaviour (16%, n=27), depression (6%, n=11), hallucinations/delusions (6%, n=10), anxiety/crisis (4%, n=7), overdose ingestion (3%, n=5). | Study population was people presenting to ED. Observation only (38% n=65), medical interventions (62% n=108), admitted (14% n=24). |
| Myran, 2022 [47] | N/A | N/A |
| Myran, 2022a [48] | N/A | N/A |
| Wang, 2020 [30]  Wang 2017 [46] | Drowsiness/lethargy (25%, n=340), ataxia (4%, n=55), seizure/tremor (8%, n=101), nausea/vomiting (23%, n=312), agitation (10%, n=135), confusion (8%, n=112), dizziness/vertigo (8%, n=101), hallucinations/delusions (4%, n=58), tachycardia (13%, n=177), mydriasis (4%, n=47), headache (3%, n=43), dyspnoea (3%, n=35), abdominal pain (2%, n=28), coma (1%, n=16)  No effect (14.4%, n=194), minor effects (50.4%, n=667), moderate effects (15.7%, n=211), major effects (1.1%, n=15). One paediatric death in an 11 month old (presumably same case as reported in Wang 2016). | N/A |
| Bennett, 2021 [29] | N/A | Study population was hospital encounters. Inpatient (50%, n=645), emergency department (28%, n=360), observation unit (22%, n=291), ICU stay (15%, n=199), NICU stay (0.3%, n=4), mechanical ventilation (4%, n=53) |
| Beauchamp, 2018 [31] | N/A | N/A |
| Dewey, 2020 [45] | Minor effect (25%, n=14), moderate effect (18%, n=10), major effect (4%, n=2).  Symptoms only reported for the two children with major effects. One child was intubated due to CNS depression and possible seizure. Another child was intubated due to altered mental status and cardiovascular instability. | Managed at a health care facility (81%, n=46), admitted (28%, n=13), critical care admission (15%, n=7). |
| Wang, 2018 [42] | N/A | Study population was ED visits. Discharged home (51%), admitted (30%). |
| Spyres, 2015 [44] | Coma/CNS depression (15%, n=24), agitation (13%, n=20), delirium/psychosis (10%, n=16), seizure (5%, n=8), hallucination (4.4%, n=7), Hypertension (5%, n=8), hypotension (2%, n=3), tachycardia (11%, n=17), respiratory depression (2.5%, n=4). | Study population is patients in the ToxIC registry (i.e. hospitalised patients), toxicologic treatment given in 43.0% (n=68) patients. |
